# Editorial: Women in environmental toxicology

**DOI:** 10.3389/ftox.2024.1490209

**Published:** 2024-11-05

**Authors:** Laura M. Langan, Michelle Bloor, Amanda Sevcik, Erica D. Bruce

**Affiliations:** ^1^ Department of Environmental Health Sciences, University of South Carolina, Columbia, SC, United States; ^2^ School of Social and Environmental Sustainability, University of Glasgow, Glasgow, Scotland, United Kingdom; ^3^ Department of Biology, Baylor University, Waco, TX, United States

**Keywords:** environmental toxicology, gender parity, SDG5 gender equality, women in STEM fields, unconscious bias, gender bias

Women are underrepresented minorities in Science, Technology, Engineering, and Maths (STEM) fields, in addition to “leaking” from academia at higher rates than their male counterparts. At every career stage, generally irrespective of discipline, the proportion of women decreases in academia, which is a phenomenon termed the ‘leaky pipeline’ (Blickenstaff, 2005). Worldwide, 33% of researchers are women (varying substantially by country) and only 12% of national science academics are women (UN, 2024). Deepening this issue, smaller research grants are often awarded to women compared to their male colleagues (Jebsen et al., 2022) and women’s research is underrepresented in high-profile journals as they submit fewer scientific papers to top journals compared to their male counterparts (Ross et al., 2022). These issues are frequently overlooked, requiring collective visibility and capacity building to eradicate the gender gap. UNESCO has acknowledged that the gender gap is an issue that is hindering progress towards attaining the sustainable development goals (SDG), where science and gender equality are essential to ensure sustainable development.

The field of toxicology is intrinsically linked to these goals, contributing to the protection of the world population through various activities including improving public health, access to clean water, and the sustainable consumption and production of safe food. As a multidisciplinary field, environmental toxicology explores how chemicals affect human health and the environment, through principles of biology, chemistry, and epidemiology. While the exact number of female researchers in the field of environmental toxicology is unknown it can be predicted that women are underrepresented, as is the case of most STEM fields. Long-standing biases and gender stereotypes, accumulated over decades, are discouraging girls and women away from science-related fields. This Research Topic represents a timely, if not the first, platform for the promotion of women scientists across this subfield and joins Research Topic highlighting women-led research in toxicology (Bryant-Friedrich et al., 2023), regulatory toxicology (Ågerstrand et al., 2022), biosensor research (Cristea, 2023), nanotechnology (de Bettencourt-Dias and Hahm, 2018), in addition to physics (Ponce Dawson, 2023), and glycoscience (Roman et al., 2023).

As part of a drive to highlight female leadership, a recent new phenomenon, we wish to highlight that the authors of the papers within the Research Topic are at various stages of their scientific careers, from doctoral students to established leaders, working around the world with a common goal. Representing a snapshot of women-led science, the papers herein provide a glimpse of some of the recent and diverse contributions, made by women, to the field of environmental toxicological sciences. We proudly present this Research Topic, and we hope that it will inspire the next-generation of female scientists while also offering some thoughts on how to dismantle and lower the real and perceived barriers within STEM. The five contributions, written by 18 authors of which 12 are women, include 5 original research articles, and 1 review, and all the papers explore different aspects of environmental toxicology.

The first, second and fourth article provide important information from both field and laboratory studies to feed into ecological risk assessments. Focusing on fish, Rojo et al., provides important information about the bioconcentration kinetics to human pharmaceutical active ingredients in several understudied neotropical fish species in Brazil, while Jeninga et al., contributes to our understanding of chronic exposure to mixtures of neonicotinoid insecticides in fathead minnow larvae. In Pihlaja et al., 16 human pharmaceuticals in different therapeutic classes were examined for P450 inhibition via CYP1A and CYP3A-activity using the rainbow trout liver S9 fractions, adding important toxicological information for which environmental effects and fate data is currently very limited. The third article written by Cheng et al., highlights the association between ambient air pollution and the occurrence of thyroid cancer. The study used certified diesel particulate matter as a proxy for fine particulate matter to expose and examine functional response using human thyroid cancer cell lines. Their results suggest that fine particulate matter induces augmented collective cell migration in thyroid cancer cells. Finally, the review article by Solan and Park, explores the current literature Per- and poly-fluoroalkyl substances (PFAS) exposure, with a specific focus on their inhalation, the adverse effects on lung health, and the mechanisms underlying tissue- and cellular-level adverse outcomes.

While this Research Topic highlights the diverse areas where women-led science is benefiting and enhancing our understanding of environmental toxicology, we must as editors highlight some of the difficulties encountered during the formulation, recruitment and submission process for this Research Topic, including in the timelines for submission, but also in the identification of female editors in senior positions in this field, combined with the small pool of researchers led by a female PI or have a female lead researcher. While stark, this is not surprising. Starting early (i.e. 10–12 years), multiple social, educational and personal aspects are contributing to perpetuate STEM stereotypes (Guenaga et al., 2022). Sidelined, isolated and sometimes discriminated against, women are balancing societal expectations and caring responsibilities with career progression (McKinnon and O’Connell, 2020; Carter et al., 2024), in addition to contributing to invisible work in the labour market (Kaplan, 2022).

Numerous initiatives are being carried out to address these issues including female role model mentorship programs, where children, teenagers and women are paired with role models locally, regionally and nationally are showing some beneficial outcomes. Such programs and other initiatives are available through schools (González-Pérez et al., 2020; Guenaga et al., 2022), scientific societies (e.g., Society of Environmental Toxicology and Chemistry, Society of Toxicology), higher education institutions (e.g., United Kingdom Advance Higher Education) and research councils (e.g., UK Biotechnology and Biological Sciences Research Council), and there is evidence that visibility and mentorship contributes to uptake and continuation of STEM vocations. Yet, more action is still needed to support women in STEM. Women now account for 44% of all PhD’s but only 33% of researchers, with a trend towards female graduates now opting not to pursue careers in research, an unsurprising dilemma. If we want to see more women remaining in research, effort and resources must be concentrated to achieve this goal, especially in dealing with unconscious bias.

Unconscious or implicit bias refers to the attitudes or stereotypes that affect our understanding, actions, and decisions in an unconscious manner. Early seminal research by Wennerås and Wold (1997) identified sexism, and nepotism impacts in medical research fellowship’s reviews such that for a female to receive the same competence score as a male, a female needed to exceed his scientific productivity by three extra Science or Nature papers or 20 extra papers n lower-impact professional Journals. With his field representing biology and medicine, two fields where gender balance was perceived to have attained more gender parity, this study was one of the first to document the impact of bias through the examination of genuine peer-reviewer evaluation sheets. In STEM fields, more recently, there has been some improvement in unconscious bias recognition through efforts to raise visibility and awareness, resulting in rigorous efforts to avoid it. However, there are still biases in the field of environmental toxicology that can impact women in several ways. This bias is visible in several different ways, for example, the lower number of women reaching full professor in comparison to men, grant funding, recruitment, evaluation and recognition (Larivière et al., 2013; Carr et al., 2017; Kozlowski et al., 2022; Ioannidis et al., 2023) and leadership roles within toxicology (Spyres et al., 2019; Swanson, 2022). However, also more general bias among managers which can manifest in feeling ‘judged’ or bullied and even harassed for juggling careers with home life and children as well as dealing with gender prejudice in the workplace (Cunningham and Cunningham, 2022). Saturating evaluation metrics, these biases can impact hiring and promotion, funding and research opportunities, recognition and awards, peer review and publication for academics and career progression in general across STEM fields (Carr et al., 2017; Witteman et al., 2019). Addressing these prejudices requires a multifaceted approach, including awareness training, policy changes, mentorship programs, and initiatives to support diversity, equity and inclusion (Melo, 2021; Kozlowski et al., 2022).

By recognizing and actively working against unconscious biases, using a combination of the above-mentioned approaches, the field of toxicology can become more equitable and supportive for women. To change the *status quo*, and traditional mindsets, gender equality in science must be funded, prioritized and encouraged. Promoting our fellow women colleagues, extending collaborative networks by sharing, being visible but also actively being aware of conscious and unconscious bias are just some examples of what can be done to contribute to a more diverse and inclusive workforce. Improvements have been made, but progress is slow and there are still gaps to fill. We offer our thanks to the authors and reviewers who contributed to this Research Topic, and also helped us to shine a light on the challenges faced by women in environmental toxicology and wider STEM fields.
